# Simple Model of Protein Energetics To Identify Ab Initio Folding Transitions from All-Atom MD Simulations of Proteins

**DOI:** 10.1021/acs.jctc.0c00524

**Published:** 2020-07-21

**Authors:** Massimiliano Meli, Giulia Morra, Giorgio Colombo

**Affiliations:** † SCITEC-CNR, Via Mario Bianco 9, Milano 20131, Italy; ‡ Weill-Cornell Medicine, 1300 York Avenue, New York, New York 10065, United States; § University of Pavia, Department of Chemistry, Viale Taramelli 12, Pavia 27100, Italy

## Abstract

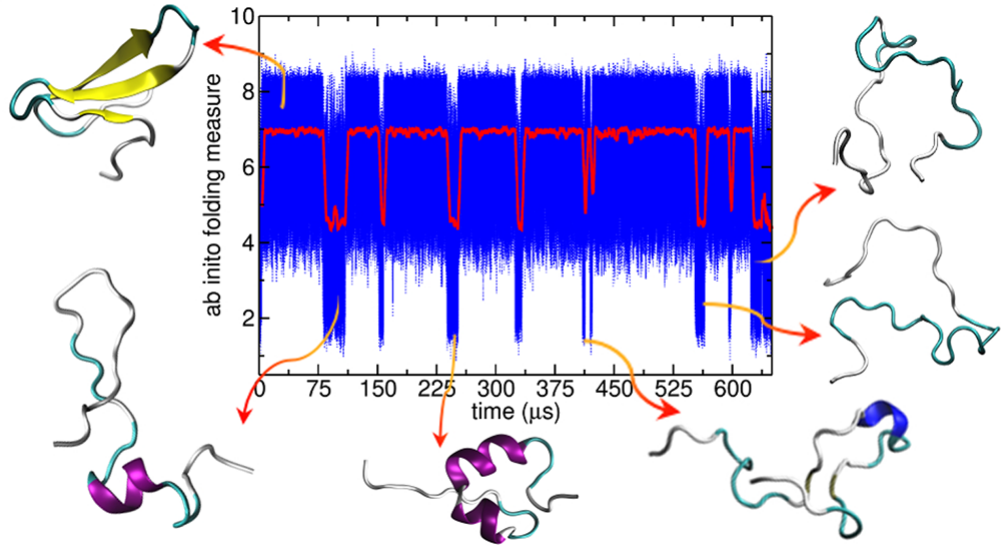

A fundamental requirement to predict the native conformation, address questions of sequence design and optimization, and gain insights into the folding mechanisms of proteins lies in the definition of an unbiased reaction coordinate that reports on the folding state without the need to compare it to reference values, which might be unavailable for new (designed) sequences. Here, we introduce such a reaction coordinate, which does not depend on previous structural knowledge of the native state but relies solely on the energy partition within the protein: the spectral gap of the pair nonbonded energy matrix (ENergy Gap, ENG). This quantity can be simply calculated along unbiased MD trajectories. We show that upon folding the gap increases significantly, while its fluctuations are reduced to a minimum. This is consistently observed for a diverse set of systems and trajectories. Our approach allows one to promptly identify residues that belong to the folding core as well as residues involved in non-native contacts that need to be disrupted to guide polypeptides to the folded state. The energy gap and fluctuations criteria are then used to develop an automatic detection system which allows us to extract and analyze folding transitions from a generic MD trajectory. We speculate that our method can be used to detect conformational ensembles in dynamic and intrinsically disordered proteins, revealing potential preorganization for binding.

## Introduction

The prediction of the native conformation of a protein of known sequence is one of the most fascinating problems in molecular biophysics.^1−4^ In recent years, the evolution of simulation techniques and computing hardware and the increase in the sophistication and resolution of experimental methods have determined a substantial convergence between the mechanistic details accessible to atomic-level simulations and those obtainable from experiments.^5−9^ In this framework, reversible folding of globular proteins of dimensions up to 100 residues has come within reach of Molecular Dynamics (MD) simulations, providing direct access to thermodynamic and kinetic quantities such as folding rates, free energies, folding enthalpies, heat capacities, and temperature-jump relaxation profiles.^10,11^ In general, all MD studies rely on the previous knowledge of the native structure of the target protein to define the folded or unfolded ensembles and the kinetics involved in the transitions between them.

Despite significant advances and success, this still leaves important questions open. First, in the absence of information on the 3D organization of the native state, can we define a simple and reliable reaction coordinate that permits one to label a certain conformational ensemble as the most likely native one? Second, considering folding a particular type of conformational transition, can we extend the use of this simple descriptor to identifying functionally relevant conformational changes, frequently occupied conformations, misfolded states? Can we obtain a reliable residue-based metrics to highlight the role of specific sites in determining such phenomena and then use this information to guide the design of new sequences of artificial proteins?

Ideally, one would need to develop a blind, automated method able to deal with the high numbers of structures visited during a folding simulation while at the same time capable to classify single snapshots as folded or unfolded. Recently, machine-learning approaches have proved able to predict the fold of a protein by relying on the knowledge of sequence alignments and the proximities between residue pairs in other proteins of known structures.^12−14^ On the other hand, physicochemical approaches have combined simulations with structural and kinetic analyses to build, e.g., Markov state models able to reproduce the main traits of folding and conformational dynamics.^15−21^

Overall, a generalized method to analyze folding transitions on long MD trajectories is required to reduce the dimensionality of the complex conformational space to its most essential (and thus treatable) traits by defining an ab initio reaction coordinate able to monitor the folding reaction and to highlight important transitions on this reduced dimension landscape.

Furthermore, an ideal reaction coordinate should give a distinctive signal characteristic of the folded state, even in the absence of previous structural information on the latter. In this context, the descriptor could be used both for the prediction of the native states of new sequences and to identify potential unfolded/misfolded states.

To progress along this route, here we build on the hypothesis that the appropriate folding reaction coordinate resides in the spectral gap of the (simplified) internal interaction energy matrix associated with a certain structure/sequence combination, which we simply call ENergy Gap (ENG). We extract structures from MD trajectories of protein folding and analyze their residue–residue pair interactions by building, for a protein of *N* residues, the *N* × *N* matrix (*M*) of nonbonded interactions (see Materials and Methods). Through eigenvalue decomposition we have previously shown that the *N* components of the eigenvector associated with the lowest eigenvalue identify residue pairs behaving as strong, stabilizing interaction centers. Furthermore, if the separation between the lowest eigenvalue and the successive one (spectral gap) is larger than the average separation among all eigenvalues, we hypothesize that the corresponding state can be defined as one of higher stability, a property distinctive of native states. This approach is called the energy decomposition method.^22−26^

**Figure 1:**
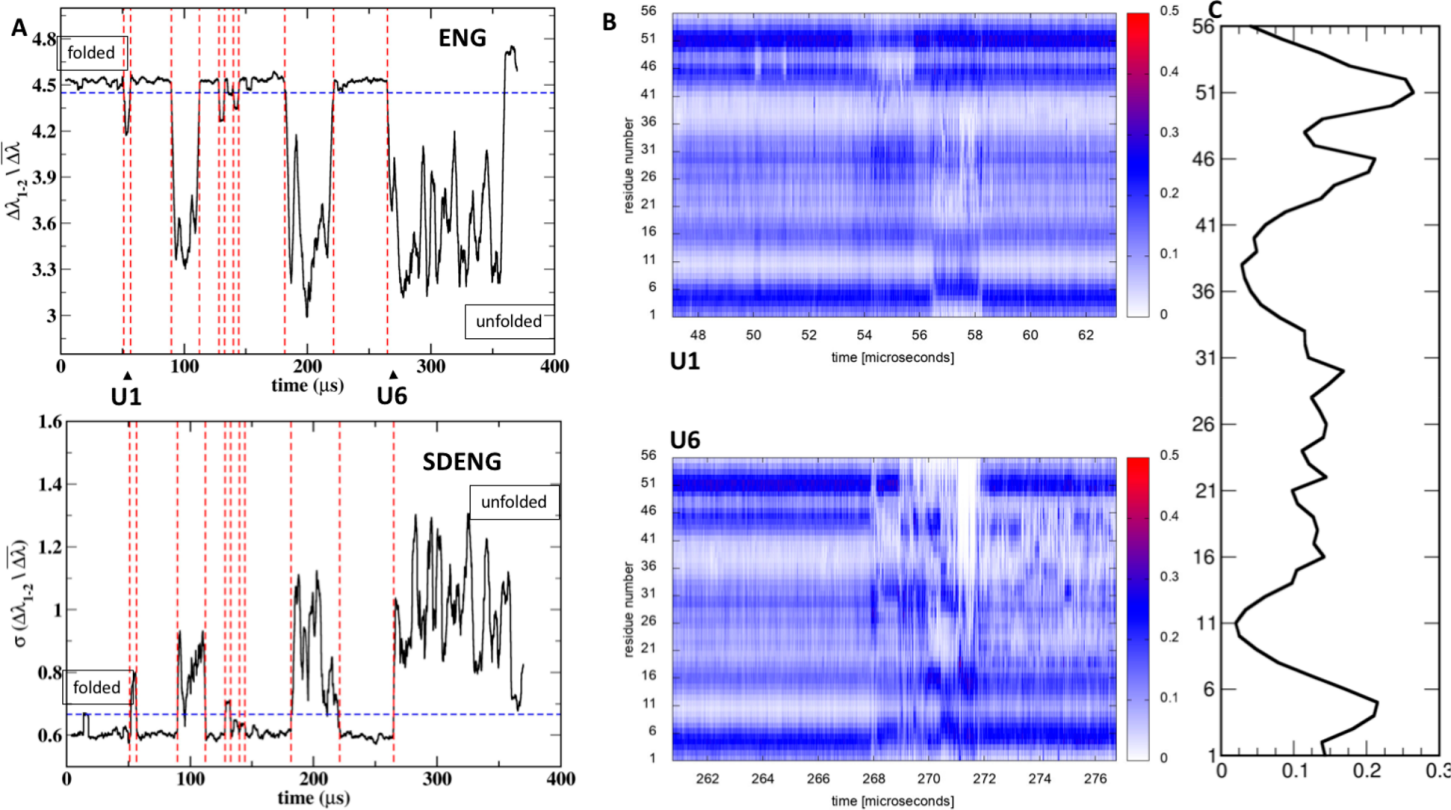
Summary of the folding detection method illustrated for a trajectory of protein G. (A) (Top) Timeline of the running average of ENG(*t*) evaluated along the trajectory: Identified threshold for ENG (see main text) is depicted in blue. (Bottom) Timeline of the running average of the corresponding SDENG(*t*) evaluated along the trajectory with threshold in blue. Detected transitions for which ENG lies above its threshold while SDENG lies below its threshold are highlighted in red in both plots. (B) (Top) For the unfolding–refolding transitions identified as U1 in A, a close up of the timeline of energy eigenvector components is plotted for each residue. (Bottom) Timeline of the energy eigenvector components during the unfolding transition identified as U6 in A. (C) Energy eigenvector of the native state of protein G is shown for comparison.

**Figure 2:**
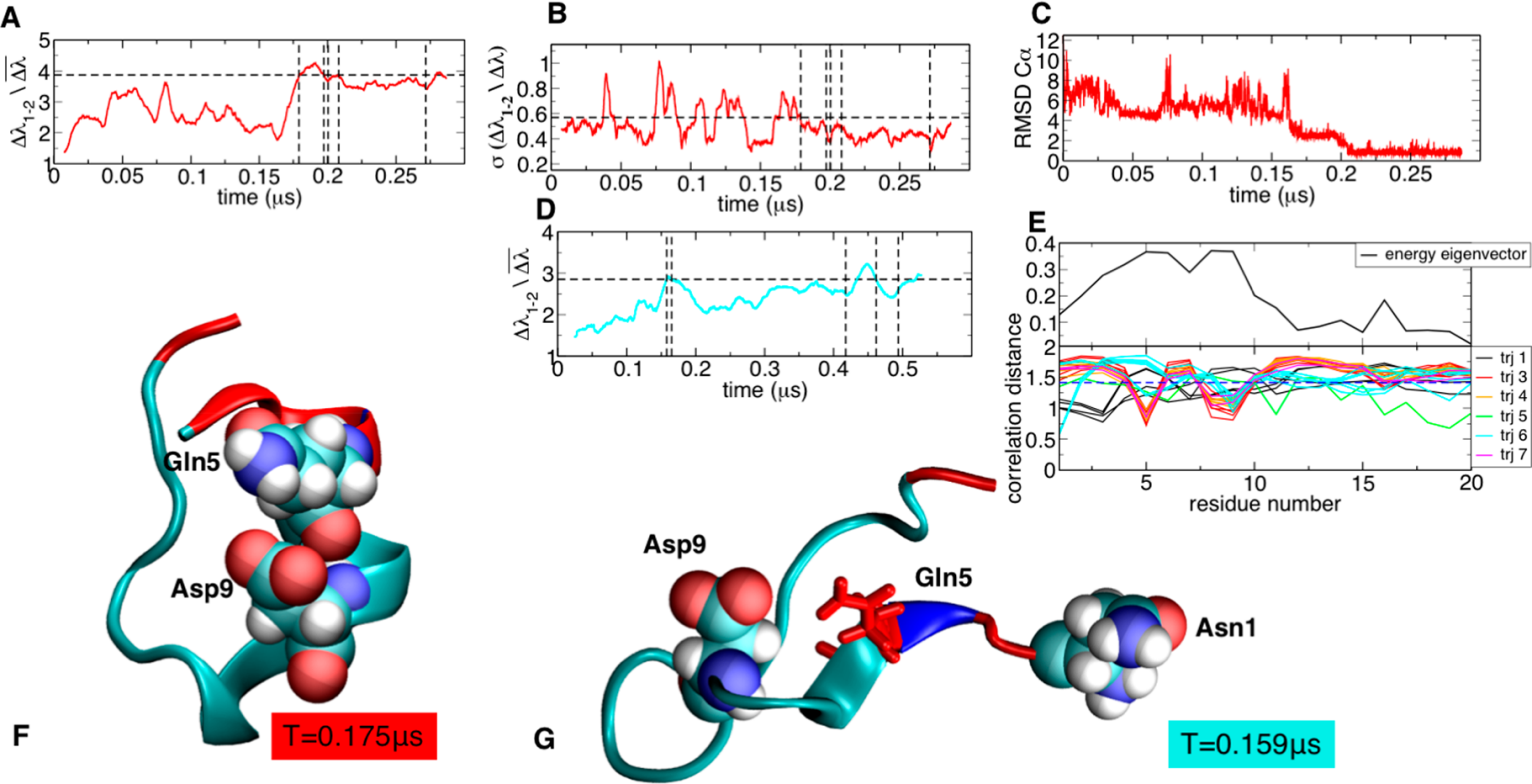
Trp cage folding analysis: (A) energy gap trajectory 3, (B) running standard deviation of energy gap trajectory 3, (C) RMSD to native structure trajectory 3, and (D) energy gap trajectory 6. Dashed lines indicate the identified transitions. (E) Profiles of the folded energy eigenvector and of the lambda–energy eigenvector correlation for each transition. (F) Folded state identified in trajectory 3. (G) Misfolded state identified in trajectory 6. Contact between Gln 5 and Asp9 is lacking the native-like backbone interaction.

Here, we select the energy matrix spectral gap ENG as the (time-dependent) parameter that captures the energetic determinants of the protein necessary to distinguish the native state from alternative ones. We apply this concept to analyze a series of micro- to millisecond long folding–unfolding trajectories of proteins of different lengths and secondary/tertiary structure contents. We show that structural basins of native states are characterized by high-energy gaps (ENG) elevated from the minimum, stable, and with low fluctuations in time (see Figures 1, 2, 4, and 5). On these bases, we develop an automated method to identify folding transitions with no prior knowledge of the native state.

We suggest that our ability to address the folding process at atomistic resolution with a simple physics-based descriptor can be important for both fundamental and practical reasons. From the fundamental point of view, being able to characterize an ensemble of conformations obtained from MD simulations as native, independently of any previous knowledge of reference structures, can further our understanding of the relationship between protein sequence, structure, and self-organization mechanisms. From the practical point of view, by increasing our understanding of the molecular-level origins of 3D structural organization, we will be able to better engineer novel sequences with characteristics suitable for specific applications.

## Theoretical Background

In this section, we aim to introduce the principal traits of the native state identification strategy based on the energy decomposition method. The specific technical details are reported in Materials and Methods.

The energy decomposition method (EDM) is a pair decomposition scheme that aims to illuminate how the stabilization energy is partitioned within the protein.^22−25^ The basic assumption of the method is that the stabilization energy is not evenly distributed within a protein structure; rather, specific patterns of interacting amino acids will concentrate most of the energy required to favor a certain 3D arrangement. These patterns can be exposed by studying the pair-interaction matrices that recapitulate the nonbonded interaction energies of proteins in MD simulations. Through eigenvalue decomposition of the matrices and analysis of their spectra, we can learn properties of the states visited, such as stabilization hots pots, effect of mutations, and perturbation of the native state.^26−33^ Here, we extend the approach to the analysis of states alternative to the folded one to include misfolded and unfolded conformations of a diverse set of proteins. We therefore focus on the pair interaction energy as well as its eigenvalue decomposition along extensive MD trajectories.

A relevant quantity in this approach is the energy gap, defined as
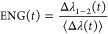
where the pair energy matrix is calculated at a given time step *t*; therefore, its eigenvalue decomposition is time dependent. We start from the observation that in simplified models of proteins the gap between the two most negative eigenvalues Δλ_1–2_(*t*) at a given time *t* is significantly larger than the average gap in the native state than in alternative states ⟨Δλ_1–2_⟩, a distinctive property of proteins compared to heteropolymers.^34^ We extend this observation to all-atom models by asking whether in a dynamic structural ensemble, where the protein explores different alternative conformational states, the stability gap might increase as the protein resides in or approaches the native state. In fact, we observe that structural basins of native states are associated with high spectral energy gaps (ENG), forming plateaus that are stable in time, and whose values are characterized by low standard deviations in energy gaps (SDENG) compared to alternative, unstable states. The basics of the method are illustrated in Figure 1.

On the basis of these observations, we develop a simple, direct, and generally applicable method to identify folding transitions with no prior knowledge of the native state. The method is based on analysis of the time evolution of the spectral energy gap (ENG) and its fluctuations (SDENG) from long folding equilibrium MD simulations (spanning time scales from microseconds to milliseconds) to identify the areas of maximal gap and minimal fluctuations in the lambda criterion as a distinctive marker of the native state and folding–unfolding transitions.

The detection system we present here is general as it does not depend on the system. Moreover, it is fully automated. Given a MD trajectory and without any knowledge of the native structure, ENG(*t*) and SDENG(*t*) are calculated along the trajectory evolution and threshold values for both time series are initialized at the maximum ENG/minimum SDENG value, respectively. Then by moving the thresholds and counting the populations above/below them, optimal threshold values to detect the transitions are found (based on the sigmoidal behavior of the populations) (see Figure 1 and Supporting Information Figure S1). After automatically defining the thresholds, the algorithm yields a list of possible folding transition intervals that can be further analyzed. Details are outlined in Materials and Methods.

### Analysis of the Transitions and Identification of Residues Critical for Folding

Calculating the energy partitioning within a protein structure along a MD trajectory not only permits one to identify folding transitions but also to directly monitor energy contributions at a single-residue level and highlight interactions relevant for folding as well as transient ones without any prior knowledge of the folded state (Figure 1B), namely, once the folding–unfolding transitions have been identified by calculating the energy gap (ENG) and its fluctuations (SDENG), the main essential interactions driving them can be identified by focusing on the *N*-dimensional energy eigenvector associated with the lowest eigenvalue (Figure 1C). The energy eigenvector^22,35^ recapitulates the contribution of each residue in a protein sequence to the stability of a conformational state with peaks representing the amino acids mostly involved in stabilizing interactions. If we focus on the time evolution of the peaks along the trajectories and specifically during the conformational transitions, we can highlight those residues whose energy component changes the most upon folding, either by increasing or by decreasing their contribution to the stabilization of the native state. We hypothesize that the former can be associated with the native contacts driving folding (folding core). The latter, on the other hand, may be non-native contacts that need to be disrupted for the protein to reach the “high-energy gap” (i.e., native) state.

To identify the two subsets, we introduce a correlation measure based on the Pearson coefficient (*p*) between two time series, collected around an identified transition, namely, the time series of ENG and the time series of each energy eigenvector component (see Materials and Methods). For every residue we calculate the metric
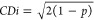
This parameter expresses a similarity measure between the two time series. We suggest that the residues that drive folding–unfolding can be identified by the high correlation (minimum *CDi* value) between their energy component and the ENG. Non-native contacts, on the other hand, decrease their stability contribution upon folding; hence, they are likely to be less correlated and lead to a high *d* value. In this framework, the regions of *maximal correlation* associated with minimal values of *CDi* (minimum distance) predict the folding core. The regions of *maximal anticorrelation* (maximum distance) correspond to residues involved in *non-native contacts that need to be disrupted* to allow the protein to proceed to the folded ensemble. Such residues are frustrated in the native state.

## Results

### Applications to Protein Simulations

The strategy described in the previous paragraphs is applied to the equilibrium atomistic simulations of different protein systems, varying in sequence length, secondary structure content, and tertiary organization. The extent of the simulations ranges from microseconds to milliseconds.

Here, we first discuss two peptides, Trp-Cage^36^ and Chignolin,^37^ which represent minimal models that had been experimentally used to gain insight into folding mechanisms of bigger proteins. Next, we set out to use our approach to identify and characterize the native states of a small protein with a mixed alpha–beta fold, BBA,^38^ and of larger proteins, including the all-α-helical A3D protein,^39^ the all-beta WW domain,^40^ and finally the mixed alpha–beta Protein G.^41^

#### Trp Cage

Trp cage (NLYIQWLKDG GPSSGRPPPS) is a 20-residue polypeptide which was shown by NMR and other biophysical techniques to fold into a short α-helix from residues 2 to 8, a 3_10_-helix from residues 11–14, and a C-terminal poly proline II helix to pack against the central tryptophan.^36^ We simulated its folding starting from a fully extended conformation using multiple independent MD replicas (see Materials and Methods for details). Figure 2A reports the time-dependent evolution of the RMSD of structures visited during one representative trajectory with respect to the native structure. In general, after visiting structures with high RMSD, the peptide collapses to the folded state, which is then populated for most of the time.

It is immediately seen that both the energy gap (ENG) and the standard deviations of the energy gap (SDENG) mirror the evolution of RMSD (Figure 2B and 2C). In the folded states (low RMSD), the protein is characterized by a larger gap between the first (most negative) eigenvalue and all others compared to alternative states together with minimal fluctuations for that state.

If the native structure were unknown, the classification of conformations using the ENG and SDENG criteria would have efficiently permitted to identify an optimal guess for the folded state.

Independent replicas (Figure 2, simulations labeled 1 and 6) show collapses to other metastable non-native states characterized by high-energy gap ENG values. Importantly, analysis of ENG evolution shows that the gap is generally lower for these non-native cases compared to those where correct folding is observed (see Figure 2A vs Figure 2D).

The ENG–eigenvector correlation distance profiles *CDi* (calculated for each trajectory) are calculated to detect residues contributing to or opposing the transition. Here, they further highlight the differences between folding and misfolding trajectories. In the case of the former, it appears that the contribution of single residues to stabilize the native state is consistently replicated in different trajectories (trajectories 3, 4, and 7 in Figure 2E). They show residues Gln5 and Asp9 as the main drivers of folding to the native state (see Figure 2F). In the case of the latter (such as trajectory 6), the correlation distance profiles highlight different sets of residues as drivers of the transitions to the non-native structures and, in particular, Asp9 interacting with Gly15 and Arg16 in different simulations. This suggests that trajectory 6 visits an intermediate that is also observed in simulations of Trp Cage carried out by others^42^ and that the ENG is able to detect such intermediate.

##### Chignolin

**Figure 3:**
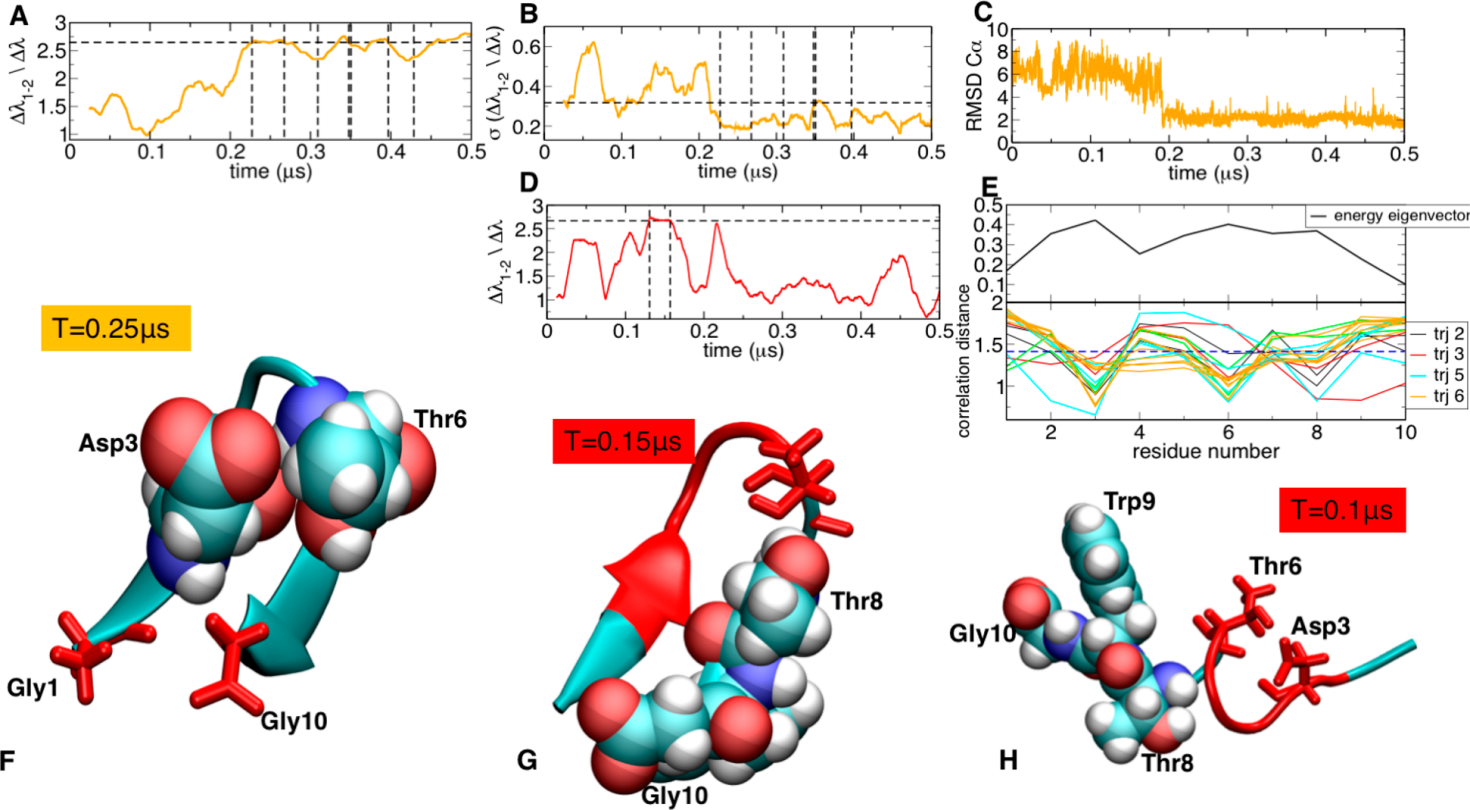
Chignolin folding analysis: (A) energy gap trajectory 6, (B) running standard deviation of energy gap trajectory 6, (C) RMSD to native structure trajectory 6, and (D) energy gap trajectory 3. Dashed lines indicate the identified transitions. (E) Profiles of the folded energy eigenvector and of the lambda–energy eigenvector correlation for each transition. (F–H) Snapshots of folded and unfolded structures extracted from the trajectories.

Chignolin (GYDPETGTWG) is a decapeptide designed to fold into a beta-hairpin, as shown by CD spectroscopy and NMR analysis at 300 K.^37^ The same simulation protocol as used for Trp cage was used to characterize the folding of Chignolin. Application of the ENG and SDENG criteria correctly identifies the folded states along the trajectories (Figure 3A–C).

Analysis of the profiles of correlation *CDi* between ENG and the energy eigenvector for each folding transition and the comparison with the profile of the energy eigenvector of the native structure highlights that a subset of residues is consistently relevant to drive folding to the experimentally determined structure (Figure 3A and 3D).

In this picture, the initial bending of the loop around Pro is stabilized by the interaction between Asp3 and Thr6, which then promotes folding via a zip-up mechanism. These two residues appear as the maximally correlated spots in the *CDi* profile for the majority of the transitions. In a short beta-hairpin, the organization of turn interactions is conceivably the most determinant factor for preorganizing the rest of the sequence in the strands to establish the ordered interactions between the beta sheets. Interestingly, Figure 3G shows an alternative mechanism observed in trajectory 3 (Figure 3D and 3E) and involving residues 8, 9, and 10 as folding drivers and 3 and 6 as anticorrelated residues, forming non-native contacts, in contrast with the other cases. This is due to a misfolding event preceding the proper folding transition at 0.1 μs, forming a helix turn involving 3 and 6, which needs to be disrupted to reach the native state (Figure 3G and 3H).

The preliminary investigations carried out on model systems support the viability of our strategy. It is tempting to state that the results described above are particularly relevant as small peptide systems in general tend to populate numerous alternative conformations whose (real) energy differences are small and difficult to quantitate using normal force-field energies. Yet, the criteria we introduced as reaction coordinates to detect folding-related conformational transitions prove able to efficiently identify native states.

On this basis, we next moved on to extend our approach to long time scale simulations of bigger realistic protein systems. The trajectories were obtained from D. E. Shaw research and refer to the systems described in ref (11).

##### BBA

**Figure 4:**
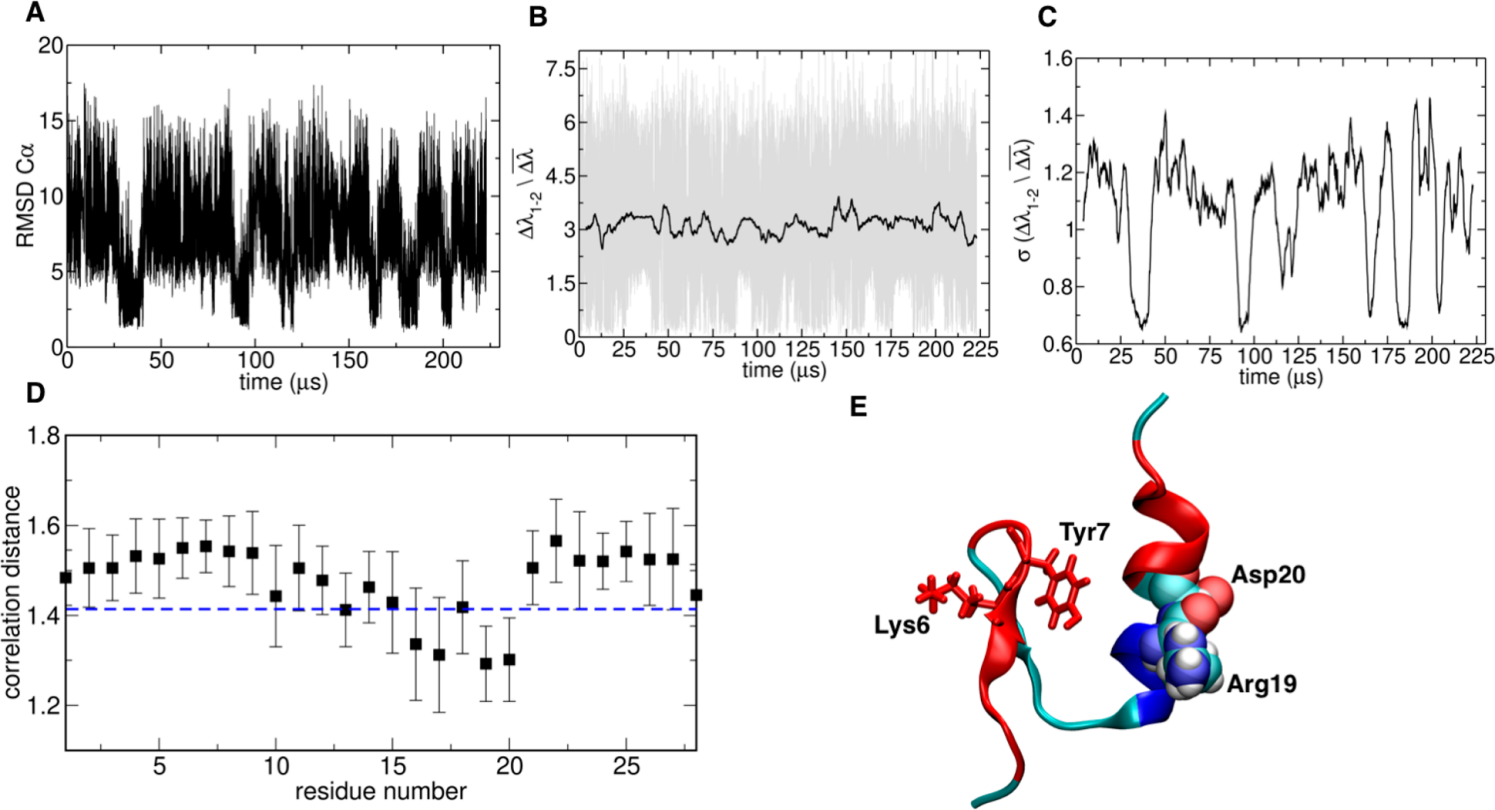
Folding–unfolding analysis of BBA: (A) RMSD to native structure, (B) energy gap, and (C) running standard deviation of energy gap; (D) average correlation distance between the energy eigenvector of each residue and the energy gap parameter, calculated over the ensemble of observed transitions. (E) Native state structure highlighting residues that have a high correlation (van der Waals and blue sticks) or a high anticorrelation (red sticks and cartoon) with the energy gap during folding–unfolding transitions.

BBA is a short sequence designed to mimic the second zinc finger of Zif268 and autonomously fold into a ββα structure without metal binding. NMR data show that the sequence reported here, 1FME.pdb, populates the desired structure, albeit with minimal stability.^38^ BBA represents thus a challenging system for our approach. The evolution of the RMSD to the native structure shows that a large fraction of unfolded conformations is present and folding events are sparse and short lived in time (Figure 4A). The ENG parameter captures the marginal stability of the system, showing relatively small gaps compared to other systems. Interestingly, the SDENG profile shows minimal fluctuations in correspondence to the minimum RMSD regions (Figure 4B and 4C).

Distance correlation *CDi* identifies the region between 16 and 20, in the α helix, as the folding core (Figure 4D). ^1^H NMR experiments indicate that the α-helix is clearly defined in the structure bundle, supporting the hypothesis that this region concentrates the maximal amount of stabilization energy. Amino acids around position 7 appear to be anticorrelated with lambda: experimental optimization of the structure indeed showed that mutations at this region significantly impact on the fold stability, modulating formation of the type I′ turn necessary to favor the correct organization of the beta-hairpin.^38^

##### A3D

**Figure 5:**
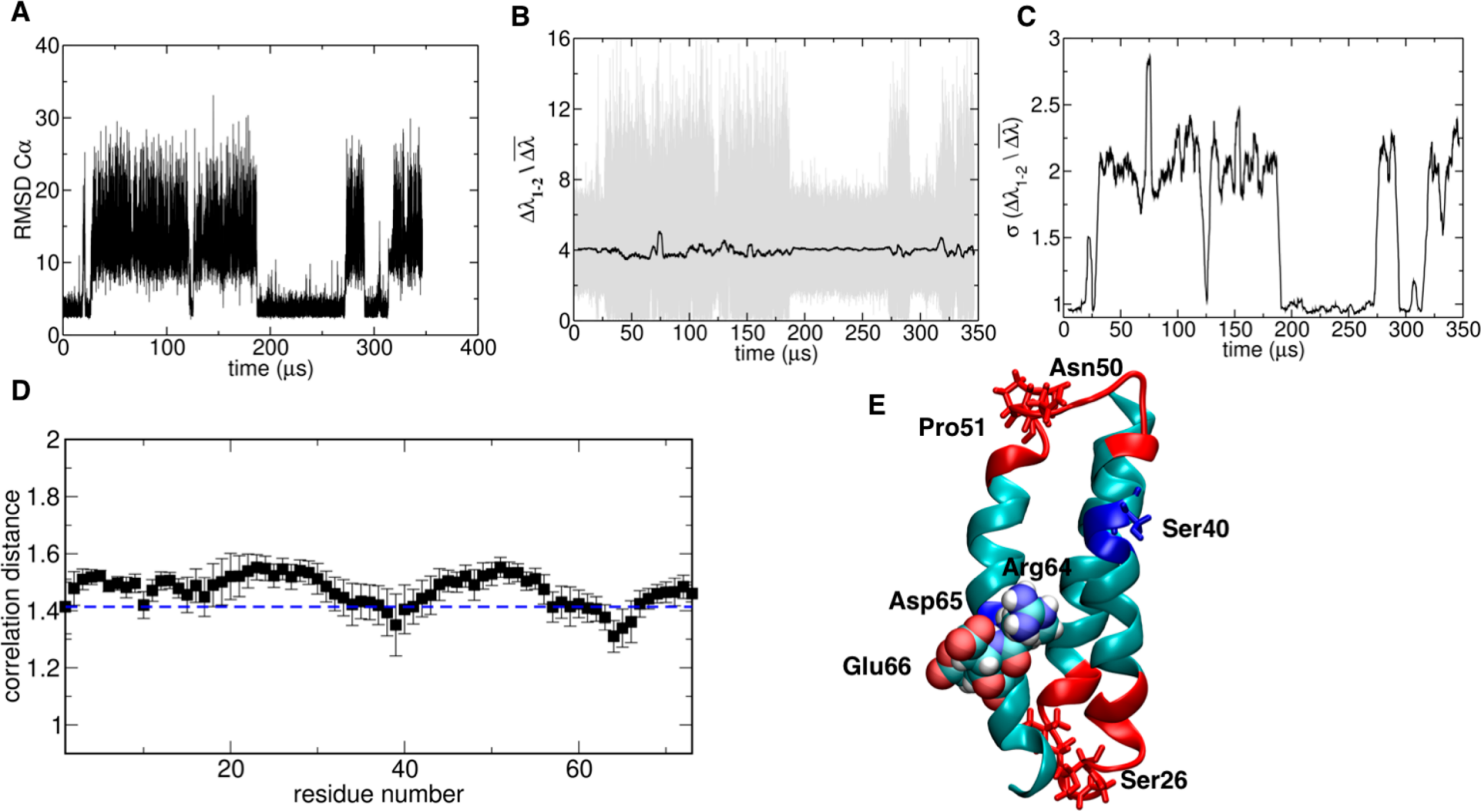
Folding–unfolding analysis of A3D: (A) RMSD to the native structure, (B) energy gap, and (C) running standard deviation of the energy gap; (D) average correlation distance between the energy eigenvector of each residue and the energy gap parameter, calculated over the ensemble of observed transitions. (E) Native state structure highlighting residues that have a high correlation (van der Waals and blue sticks) or a high anticorrelation (red sticks and cartoon) with the energy gap during folding–unfolding transitions.

Alpha3D is a designed, fast folding, 3-helix bundle protein. Folding events can be proficiently identified applying the integrated ENG and SDENG criteria (Figure 5 A–C).^39^ Investigation of the distance correlation parameter indicates that the folding core entails mainly the regions around residues 40 and 60. These regions include hydrophilic/charged amino acids (Ser40, Glu41, Arg64, Asp65, and Glu66), whose introduction has proven beneficial to favor the folding to the three-helix bundle geometry, and two hydrophobic residues (Leu42 and Leu67), whose burying from solvent can stabilize the helical structures. Interestingly, the graph shows that most of the residues in the sequence play a limited role in the stabilization of the native fold. Their interactions are thus mostly local, consistent with the observation by Lindorff-Larsen et al. that local elements of secondary structures form early on the folding pathway.^11^ The lack of strong interactions in the core of A3D is mirrored by the dynamic and variable packing observed in the folding transition state ensemble observed via experiments and calculations.^43^ The correlation distance analysis reported in Figure 5D, indicating a high fraction of residues that are anticorrelated to the spectral gap increase, is consistent with the high level of frustration reported for this protein by Clementi and co-workers.^44^

##### WW Domain

**Figure 6:**
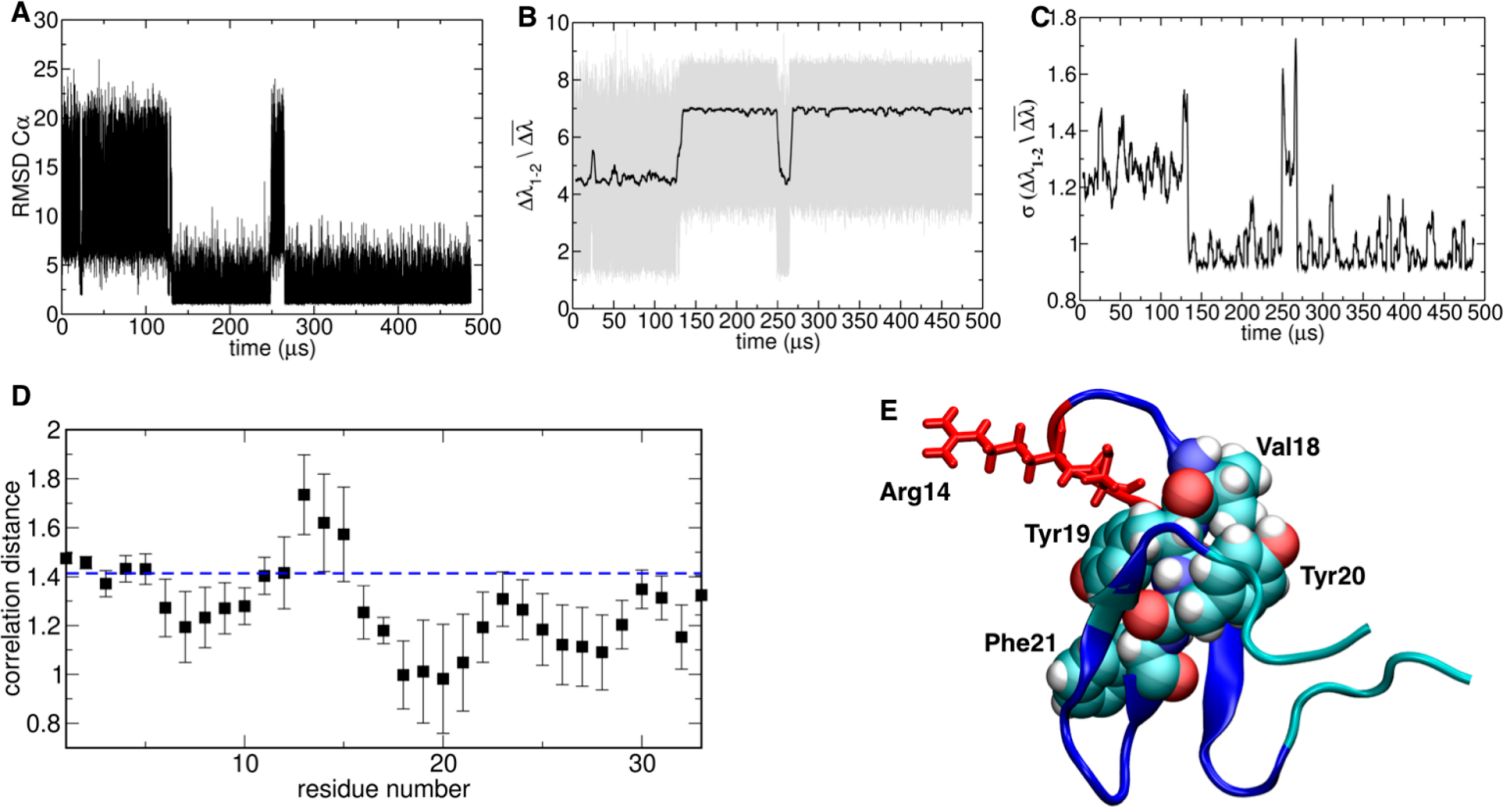
Folding–unfolding analysis of WWdomain: (A) RMSD to native structure, (B) energy gap, and (C) running standard deviation of the energy gap; (D) average correlation distance between the energy eigenvector of each residue and the energy gap parameter, calculated over the ensemble of observed transitions. (E) Native state structure highlighting residues that have a high correlation (van der Waals and blue sticks) or a high anticorrelation (red sticks and cartoon) with the energy gap during folding–unfolding transitions.

WW domains are three-stranded beta-sheet domains that are widely diffuse as interaction motifs in proteins.^40^ The time evolution of the RMSD from the reference native structure shows multiple reversible folding events, which are aptly captured by both the ENG and the SDENG analyses (Figure 6A–C). Correlation *CDi* profile shows 4 well-defined minima, scattered all along the sequence. The residues that most strongly correlate to the increase in the energy gap and stabilization of the folded state are those defining the central hydrophobic core (Figure 6D). The diffuse participation to the increase of the energy gap favoring the native state is consistent with the low levels of frustration (also compared to A3D) reported by Clementi and co-workers. Residue 30 is less correlated, suggesting that it might have significant interactions also in the unfolded state and be less critical for folding.^44,45^

##### Protein G

**Figure 7:**
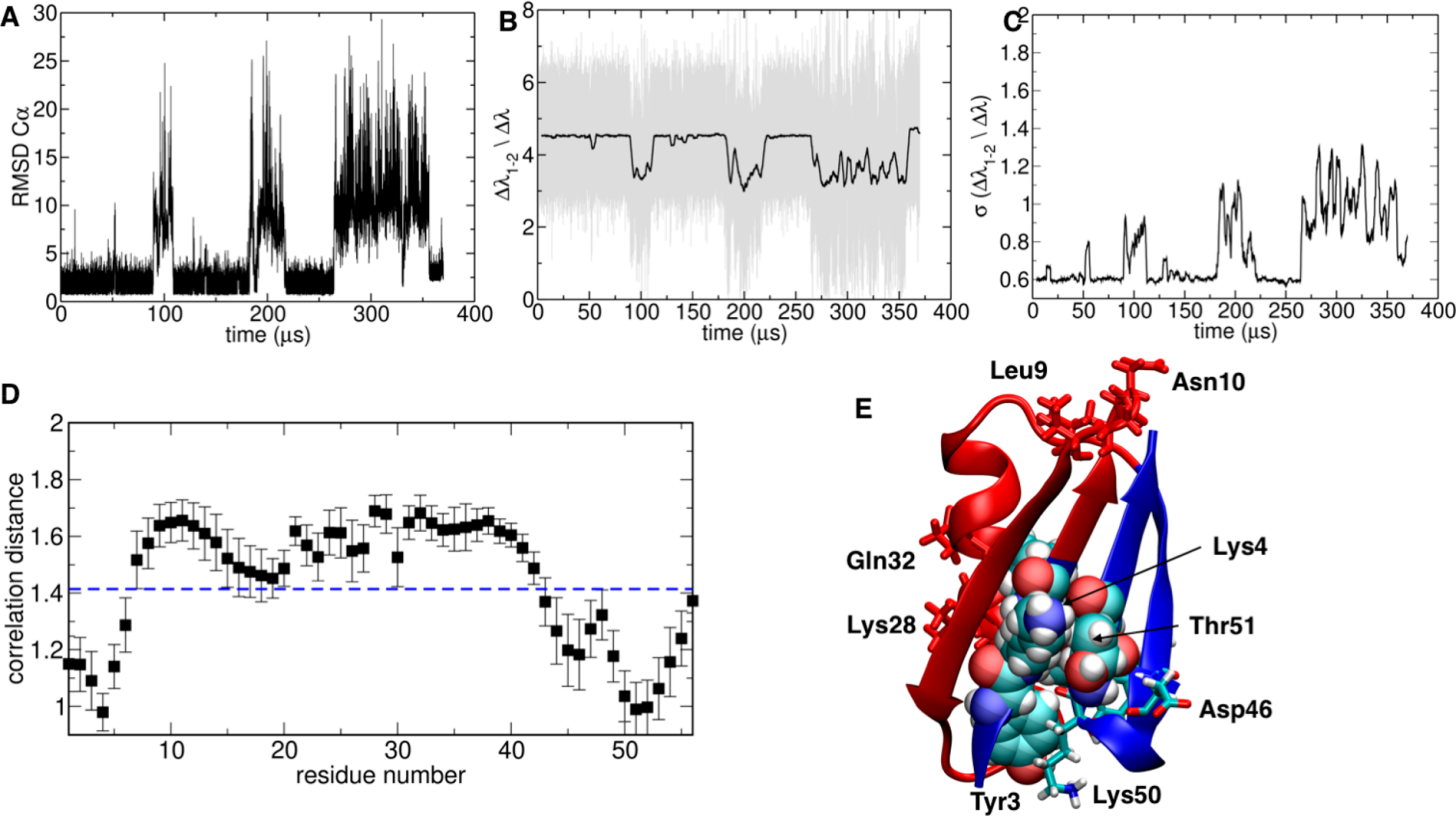
Folding–unfolding analysis of protein G: (A) RMSD to native structure, (B) energy gap, and (C) running standard deviation of energy gap; (D) average correlation distance between the energy eigenvector of each residue and the energy gap parameter, calculated over the ensemble of observed transitions. (E) Native state structure highlighting residues that have a high correlation (van der Waals and atom-named colored sticks) or a high anticorrelation (red sticks and cartoon) with the energy gap during folding–unfolding transitions.

Finally, we apply our strategy to the study of Protein G,^41^ a mixed alpha–beta protein, whose folding has been widely investigated by mutational, biophysical, and structural approaches. Figure 7A–C shows a striking correlation between the evolution of the protein RMSD and the time evolution of the ENG and SDENG reaction coordinates. Specifically, in terms of energy gap, a strong separation between energy states appears in correspondence of the folded states, paralleled by very low fluctuations.

Analysis of the correlation distances in correspondence of the various transition events sampled during the simulations highlights which residues are key to stabilize native (native-like) conformations on the folding funnel. The amino acids determining folding to the correct native structure are mostly located in the N-terminal stretch at positions 1–6 and in the C-terminal region at positions 42–52. Experimental studies have shown that intramolecular interactions involving the second beta hairpin give a strong stabilizing contribution of electrostatic origin to the folded state. Packing of the second beta hairpin against the N-terminal sequence determines further stabilization of the native state,^46^Figure 7D and 7E.

Consistent with these observations, beta-hairpin 2 has been previously observed to be able to fold in isolation, representing an independent folding unit (foldon). In contrast, the distance correlation indicates that the α-helical region, turn 1, and the first beta hairpin appear to be anticorrelated with the energetic descriptors, indicating that they may be involved in interactions that oppose correct folding. Such interactions need to be disrupted and reshaped to evolve toward the native state. Experimentally, the correct formation of turn 1 has been defined as one of the requirements for folding to the native state.^46^

## Discussion

Proteins and enzymes oversee all mechanical and chemical processes within cells. In recent years, the emergence of advanced genome manipulation techniques^47^ and the advent of directed evolution methods have spurred the development of new proteins with unprecedented properties as materials or enzymes that are capable of carrying out non-natural reactions in mild conditions, providing attractive alternatives to the use of solid-phase or homogeneous chemical catalysts.^48−51^

Functions are determined by the proteins’ three-dimensional shapes and conformational dynamics, which are ultimately defined by amino acid sequences. The diversity of protein structures revealed by crystallography, NMR, and more recently CryoEM has made the definition of simple rules connecting sequence to structure a highly challenging task. Computational approaches of very different nature have been applied to solve the folding problem, ranging from a statistics-based method, to sequence-coevolutionary analysis, to knowledge-based potentials. Very recently, breakthrough results have been obtained by applying deep-learning techniques.^12^ In this context, the improvements in force-field quality and the progress in software and hardware for molecular dynamics simulations have made it possible to study the process of protein folding on real systems in realistic time scales at atomic resolution.

In this paper, we have built on the latter observation to develop a simple, physics-based approach that permits one to detect the native states of proteins. The approach we have presented requires no previous knowledge of the 3D structure of the protein under exam or of proteins with similar amino acid sequences that can be used as starting points for modeling.

By analyzing the spectral energy gap characteristics of the various structures visited by a sequence in its dynamic evolution between folded and unfolded states, we observe that native basins are associated with high spectral energy gaps (ENG) coupled to low values of standard deviations (SDENG) of energy fluctuations. This represents a consistently conserved property of native states compared to alternative ones. In the paper, this criterion was verified for a number of peptides and proteins of variable length and secondary structure content. If the method were to be applied to systems of unknown 3D structure, the candidate structures with the highest probability of representing the native state would naturally be those complying with the two above-mentioned criteria. The problem would be then reduced to how extensive the MD simulation would be and to the quality of the force field. As force fields, simulation software, and hardware (GPUs, CPUs, ARM architectures...) are constantly improving, it is tempting to suggest that the problem of exploring conformational landscapes will be largely alleviated.

The increase in the energy gap ENG is reminiscent of the notion of connectivity in graph theory. Indeed, the ENG defined here, calculated from the decomposition of the pair-interaction energy matrix, is conceptually similar to the spectral gap of the Kirchoff or Laplacian matrix calculated from the contact matrix of the protein (see the calculation method and data in the Supporting Information and in Figure S2). In the eigenvalue decomposition of the Kirchoff matrix, while the first eigenvalue is always zero, the second one reports on the connectivity of the graph. The corresponding eigenvector can be used to separate connected subgraphs: in other words, the components with equal signs define subgraphs, or cores, that are highly internally connected and mutually less connected with other subgraphs.^52^ In our work, we observe that upon folding the spectral gap is correlated to the ENG energy gap and signals a sudden increase of connectivity (see Supporting Information). In light of the similar structure of the Kirchoff matrix and of the pair energy matrix, it is fair to hypothesize that residues involved in high-energy interactions stabilizing the native state are also responsible for increasing the connectivity^53^ and the establishment of patterns of higher connectivity is related to formation of stable subdomains.

In general, protein folding is an ensemble of conformational transitions.^2^ This observation opens up several possible new avenues for future development. On one hand, one could in principle apply the ENG and SDENG criteria to the characterization of complex functionally oriented structural changes in large/multidomain proteins involving local unfolding events and metastable states. On the other hand, once the folding core and folding mechanisms have been identified for a globular protein, knowledge of which residues are correlated or anticorrelated to folding can be used to target the evolution of mutants (with site-directed or directed evolution methods) to regions that should not negatively impact the ability of the sequence to populate the native and functional conformational ensemble.

Finally, our method can expectedly be used to detect conformational ensembles in dynamic and intrinsically disordered proteins, shedding light on their stability and preorganization for binding to receptors for function.

## Materials and Methods

### Molecular Dynamics Simulations

Simulations for TRP-Cage^36^ and Chignolin^37^ were started from a completely extended conformation. The sequence and the reference folded conformation were taken from the PDB database code 1L2Y for TRP-Cage and 1UAO for Chignolin.

TRP-Cage and Chignolin were modeled and simulated via Molecular Dynamics (MD) using the AMBER 16 suite of programs^54^ with the TIP3P water model,^55^ an octahedral water box containing 39 768 atoms (TRP-Cage, 304 protein atoms) and 15 395 atoms (Chignolin, 138 protein atoms) and CUDA implementation for GPUs. Each simulation started with an unrestrained minimization consisting of 1000 steps of steepest descent followed by 1000 steps of conjugate gradient minimization. The minimized systems were then equilibrated at 300 K for 5 ns using Langevin coupling with gamma equal to 1 ps^–1^. After this step, the relaxed systems were simulated in the NPT ensemble at 1 atm using Berendsen coupling algorithms.^56^ The full particle-mesh Ewald method was used for electrostatics.^57^ The SHAKE algorithm was used to constrain all covalent bonds involving hydrogen atoms.^58^ A 2 fs time step and a 10 Å cutoff were used for truncation of the van der Waals nonbonded interactions. Each production run has a different simulation time, ranging from 280 to 630 ns, but the same simulation temperature: 300 K, see summary in Table S1. Trajectory frames were saved every 5 ps, and the striding for the energy analysis was 25 ps. Six replicas for Chignolin and 7 for TRP-Cage were produced.

The folding simulations for proteins ProteinG, WW Domain, BBA, and A3D were provided by the D.E. Shaw Research group.^11^ We used a frame every 400 ps for all to the analyses done on these data sets.

The structural properties, such as RMSD, were calculated with AMBER. VMD was used for visualization.^59^

### Energy Calculations

Before any energy calculation, the selected snapshots were minimized for 500 steps of steepest descent followed by 500 steps of conjugate gradient. The energy calculation was then performed using the energy decomposition method, EDM, developed in our group.^22,34,60^

EDM is based on the calculation of the interaction matrix *M*_*i*j_, which is determined by evaluating the inter-residue, nonbonded interaction energies (consisting of the van der Waals and Coulombic terms) between residue pairs in a given protein conformation. The underlying assumption about excluding the intraresidue couplings in the calculation is that they do not significantly depend on the protein tertiary conformation, while the inter-residue coupling energy is modulated by the structure. The shielding effect on the electrostatic interactions due to solvent is taken into account by adding a GBSA term to the energy decomposition scheme.^61^

For a protein of *N* residues at time step *t* of the trajectory, this calculation yields an *N* × *N* matrix of pair couplings *M*_*ij*_(*t*) such that the total inter-residue nonbonded energy of the protein *E*(*t*) is given by the (half) sum over the matrix entries. The spectral analysis for this *N* × *N* matrix gives *N* eigenvectors and *N* eigenvalues
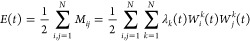
From the eigenvalues it is possible to calculate the (time-dependent) energy gap (ENG) as follows
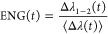
The values of ENG, calculated for each selected time frame, as well as the corresponding eigenvectors from the spectral analysis were saved and analyzed as explained in the next section.

Structure minimization and GBSA energy calculation of the interaction matrix were carried out in parallel using the gnuparallel solution (https://zenodo.org/record/1146014); the MKL Intel libraries are used for spectral analysis on the energy matrix.

### Data Analysis: Application of the ENG Criterion

As shown in the main text, the RMSD relative to the native state appears to be correlated to the ENG profile. Moreover, the fluctuations of ENG in the native state are relatively lower than in the unfolded state. From this point of view, the folded state is characterized as the state with the maximum ENG and with the minimum deviation around the mean standard deviation of energy gap (SDENG).

We used these two criteria to set up a general, automatic detection method to extract from a MD trajectory possible folding transitions without any previous knowledge of the native state.

The algorithm uses two time series as input, namely, ENG(*t*) and the energy eigenvector. On the basis of solely this information, it predicts the putative folded conformations, the F ⇔ U transitions, and the residues mostly involved in the native-like interactions associated with folding.

The code was built within the statistical environment R adding two nonstandard packages: TTR for the running averages (RA) for statistical values (mean and standard deviation) and TSclust to compute dissimilarities based on the estimated Pearson’s correlation of two given time series.

The algorithm relies on defining suitable thresholds in an automatic fashion and then selecting the folding transitions based on those thresholds. It can be separated in 3 parts: (1) choice of the running average window size, (2) choice of the threshold for mean and standard deviation, and (3) correlation calculation. They are detailed as follows.

(I) A running average (RA) step is introduced to smooth the profile of the ENG time series (see Figures 1–3, panels B and E, solid black line). The same window size is used to calculate the standard deviation SDENG profile. The window size should be large enough to reduce the noise but at the same time preserve the important transitions. Selection of the optimal window size is carried out by progressively increasing the window from a starting size (1% total number of frames) by amounts of 0.5%. At each window size, we keep track of the minimum for SDENG and the maximum of average ENG. The two resulting ENG and SDENG curves, as functions of the window size, reach a plateau value at some point, which is selected as the optimal window size.
(II) The ENG(*t*) and SDENG(*t*) curves are calculated over the running window. The second step in the algorithm consists of finding the thresholds *T*_ENG_ and *T*_SDENG_ of ENG and SDENG, respectively, such that the protein is predicted to be folded when ENG is above *T*_ENG_ and at the same time SDENG is below *T*_SDENG_. The threshold on the standard deviation in particular allows us to distinguish between fluctuations due the protein breathing motion or unproductive transitions and the proper folding (F)–unfolding (U) transitions we are looking for. In order to find the thresholds, first, we extract from the ENG and SDENG profiles the global maximum ENGmax for ENG, the global minimum standard deviation SDENGmin for SDENG, and the SD at ENGmax (Msd). Then we count the number of conformations that have ENG between (ENGmax, ENGmax – *n* × Msd) and the number of conformation that have SDENG between (SDENGmin, SDENGmin + *n* × Msd), where *n* is a % from 1 to 100. In that manner, we find our thresholds inside the uncertainty of the ENGmax. The curves drawn by these equations show a sigmoidal behavior, and the two thresholds are chosen in the proximity of the main inflection point.
(III) Once the folding–unfolding transitions have been identified, the algorithm proceeds with the analysis. Within each transition, covering an interval of twice the running average window, we focus on the changes in the components of the energy eigenvector during time (an example can be seen in Figure 1 B). The time series of each component can be compared to the ENG time series on the same interval to evaluate correlation. We calculate a correlation distance based on the Pearson’s correlation between the two time series

Correlation Distance_i_ = where *ρ*_*i*_ denotes the Pearson’s correlation between lambda and the single component of the eigenvector *V*_*i*_(*t*) during the time step
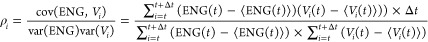
This results in a residue-based profile that contains information on the residues maximally correlated and anticorrelated with the folding transition.

## Supporting Information Available

Calculation of the Kirchoff matrix and spectral gap; example of population curve of MD snapshots above (below) the threshold vs the percentage of decrease (increase) from the starting threshold; spectral gap profile for trajectories shown in Figures 2, 5, and 7; summary of simulations for Trp Cage and Chignolin (PDF)
