# Supplementary material for: Simple Model of Protein Energetics To Identify Ab Initio Folding Transitions from All-Atom MD Simulations of Proteins
Source: J Chem Theory Comput. 2020 Jul 21;16(9):5960–71. doi: 10.1021/acs.jctc.0c00524 (PMC8009504; doi:10.1021/acs.jctc.0c00524)
Supplement: Supplementary file 1 — ct0c00524_si_001.pdf [file ct0c00524_si_001.pdf]

Supplementary material

Laplacian matrix and spectral gap

We compare the behavior of ENG during the molecular dynamics with the spectral gap calculated for the Laplacian matrix. We build a Laplacian matrix L(t) for every selected frame at time (t) of the analyzed trajectories using the formula:

$$\mathbb{L}(t) = \mathbb{D}(t) - \mathbb{A}(t)$$

Where $\mathbb{D}(t)$ is a NxN diagonal matrix that keeps on its trace the total number of contacts made by the Nth amino acid at time t. The NxN matrix $\mathbb{A}(t)$ is the contact/adjacent matrix describing residue-residue contacts. Two residues are considered in contact if their Cα distance is less than 6 Angstroms (Å), hence A(t) is defined as:

$$\mathbb{A}(t) = \begin{cases} 1 \; if \; d_{ij} \leq 6 \text{ Å} \\ 0 \; if \; d_{ij} > 6 \text{ Å} \end{cases}$$

For each time step we calculate the Laplacian matrix and its eigenvector - eigenvalue decomposition. The second eigenvalue is the spectral gap. A timeline plot of this parameter can be seen in Figure S2.

References

Chung, Fan (1997) [1992]. Spectral Graph Theory. American Mathematical Society. ISBN 978-0821803158.

Mohar, B. "The Laplacian Spectrum of Graphs."In *Graph Theory, Combinatorics, and Applications, Vol. 2: Proceedings of the Sixth Quadrennial International Conference on the Theory and Applications of Graphs held at Western Michigan University, Kalamazoo, Michigan, May 30-June 3, 1988* (Ed. Y. Alavi, G. Chartrand, O. R. Oellermann, and A. J. Schwenk). New York: Wiley, pp. 871-898, 1991.

Supplementary Figures

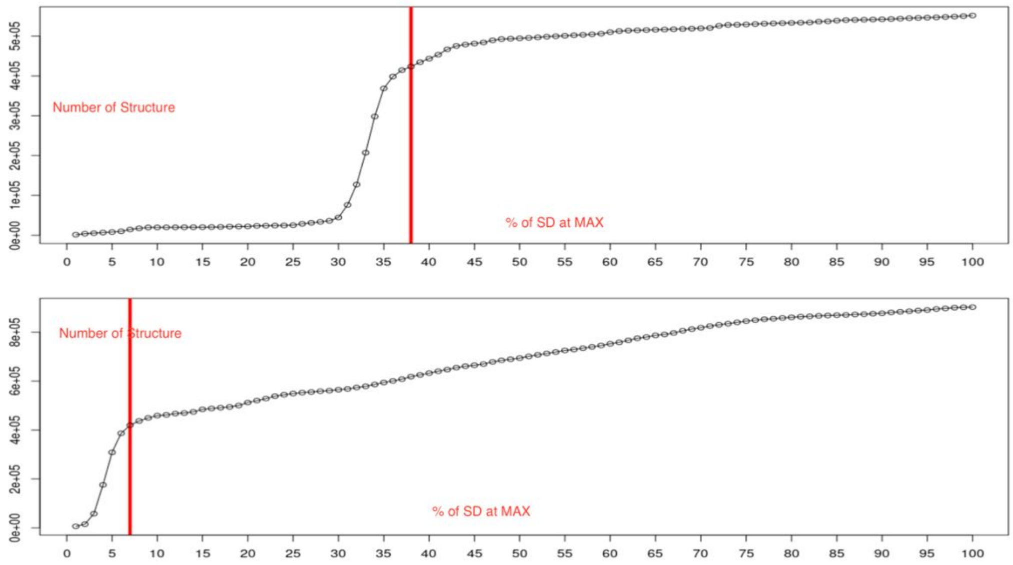

Supporting Information Figure S1: Example of population curve of MD snapshots above (below) threshold vs percentage of decrease (increase) from starting threshold. The decrease/increase steps are percentage multiples of the SD evaluated at ENGmax. Top. Number of conformations between ENGmax and [ENGmax – % of SD at MAX]

Bottom. Number of conformations between SDENGmin and [SDENGmin + % of SD at MAX].

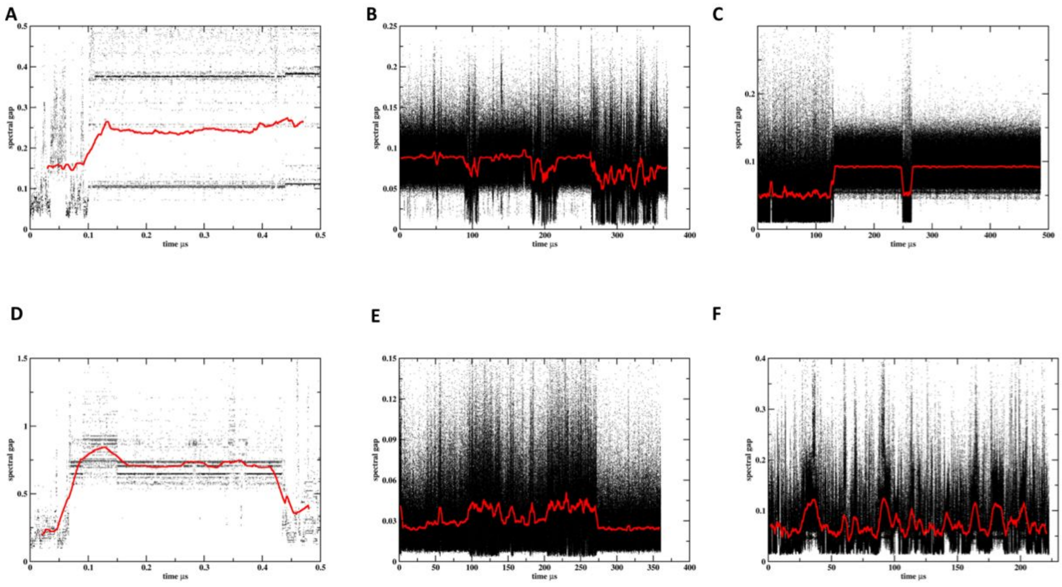

Supporting Information Figure S1. Spectral gap profile for trajectories shown in Figs 2, 5, 7 . A) TRP-Cage; B) G protein C; C) WW domain; D) Chignolin E) A3D. F) BBA. See Supplementary Material for details about calculation of the spectral gap.

Supplementary Material Table S1. Summary of TRPCage and Chignolin simulation runs

TRPCage simulation Nr	Length (ns)	Folded state reached	Chignolin simulation Nr	Length (ns)	Folded state reached
1	483	N	1	500	Y
2	413	N	2	500	Y
3	286	Y	3	500	Y
4	500	Y	4	500	Y
5	634	Y	5	500	Y
6	500	N	6	500	Y
7	500	Y			
